# Insecticide-Mediated Apparent Displacement between Two Invasive Species of Leafminer Fly

**DOI:** 10.1371/journal.pone.0036622

**Published:** 2012-05-25

**Authors:** Yulin Gao, Stuart R. Reitz, Qingbo Wei, Wenyan Yu, Zhongren Lei

**Affiliations:** 1 State Key Laboratory for Biology of Plant Diseases and Insect Pests, Institute of Plant Protection, Chinese Academy of Agricultural Sciences, Beijing, People’s Republic of China; 2 Center for Medical, Agricultural and Veterinary Entomology, Agricultural Research Service, United States Department of Agriculture, Tallahassee, Florida, United States of America; U. Kentucky, United States of America

## Abstract

**Background:**

Closely related invasive species may often displace one another, but it is often difficult to determine mechanisms because of the historical nature of these events. The leafmining flies *Liriomyza sativae* and *Liriomyza trifolii* have become serious invasive agricultural pests throughout the world. Where both species have invaded the same region, one predominates over the other. Although *L. sativae* invaded Hainan Island of China first, it recently has been displaced by the newly invasive *L. trifolii.* We hypothesized that differential susceptibilities to insecticides could be causing this demographic shift.

**Methodology/Principal Findings:**

Avermectin and cyromazine are the most commonly used insecticides to manage leafminers, with laboratory bioassays demonstrating that *L. trifolii* is significantly less susceptible to these key insecticides than is *L. sativae*. In trials where similar numbers of larvae of both species infested plants, which subsequently were treated with the insecticides, the eclosing adults were predominately *L. trifolii*, yet similar numbers of adults of both species eclosed from control plants. The species composition was then surveyed in two regions where *L. trifolii* has just begun to invade and both species are still common. In field trials, both species occurred in similar proportions before insecticide treatments began. Following applications of avermectin and cyromazine, almost all eclosing adults were *L. trifolii* in those treatment plots. In control plots, similar numbers of adults of the two species eclosed, lending further credence to the hypothesis that differential insecticide susceptibilities could be driving the ongoing displacement of *L. sativae* by *L. trifolii*.

**Conclusions/Significance:**

Our results show that differential insecticide susceptibility can lead to rapid shifts in the demographics of pest complexes. Thus, successful pest management requires the identification of pest species to understand the outcome of insecticide applications. These results further demonstrate the importance of considering anthropogenic factors in the outcome of interspecific interactions.

## Introduction

Several species of leafmining flies in the genus *Liriomyza* have become major invasive pests around the world. Two of the most economically important species are *L. sativae* Blanchard, *L. trifolii* (Burgess). The damage created by these highly polyphagous species comes from the mining of foliage by the larvae and puncturing of foliage by females for feeding and oviposition 1,2.

With the increasing international movement of horticultural products, these species have now invaded many different agricultural areas of the world. Often both species have been introduced into the same area, although, generally, at different times. However, the coexistence of the two species in invaded habitats has not been stable, with one species generally displacing the other within a relatively short period of time [3]. In California (USA), *L. sativae* was quickly displaced by *L. trifolii* following the latter species introduction in the late 1970’s [4]. In contrast, *L. sativae* has replaced *L. trifolii* after its introduction into Japan in 1999 [5], [6]. The causes of these demographic changes are not always clear, but maybe attributable to various biological mechanisms or anthropogenic factors, including differential reproductive success or susceptibility to insecticides.

In China, *L. sativae* was found first on Hainan Island in October 1993, and spread throughout China within a few years. By 2005, the distribution of *L. sativae* included more than 30 provinces throughout China [7]. Soon after its introduction, it had become one of the most important horticultural pests in the country 8,9. *Liriomyza trifolii* is a more recent invasive species in China that was first recorded in Guangdong Province and on Hainan Island in 2005 and 2006, respectively.

Since its initial discovery, *L. trifolii* has become the predominant leafminer on Hainan Island. Gao et al. [10] recently suggested that differential susceptibility to commonly used insecticides may account for the replacement of *L. sativae* by *L. trifolii*. The two most commonly applied insecticides for management of leafminers on Hainan Island are avermectin and cyromazine [10]. Both of these insecticides have translaminar properties so that they are toxic to larvae [11]. Avermectins are neurotoxins and cyromazine is an insect growth regulator, which may also inhibit eclosion of puparia [12], [13]. To determine differential susceptibility to these commonly used insecticed could account for observed changes in leafminer demographics we conducted a series of laboratory, greenhouse and field studies comparing the responses of *L. sativae* and *L. trifolii* to avermectin and cyromazine treatments.

## Results

### Larval Bioassays

Probit analyses showed that avermectin was more toxic cyromazine to both species (Table 1). These analyses further demonstrated that lethal concentrations for both insecticides were significantly higher for *L. trifolii* than for *L. sativae.* Although there were significant differences in lethal concentrations, based on non-overlapping confidence intervals, between the species for both insecticides, the differences were more pronounced for avermectin compared with cyromazine. When comparing the ratio of LC_50_ estimates, *L. trifolii* was 70.0 times less susceptible to avermectin than was *L. sativae,* and 42.4 times less susceptible when comparing the ratio of the LC_90_ estimates (Table 1).

**Table 1:** Susceptibility of *Liriomyza sativae* and *L. trifolii* to avermectin and cyromazine in leaf dip bioassays.

Species	*n*	χ*^2^*	df	*P*	Slope ± SE	LC_50_ (ppm)	95% CI	SR_50_ a	LC_90_ (ppm)	95% CI	SR_90_ a
						Avermectin					
*L. sativae*	420	8.79	4	0.067	1.84+0.31	0.564	0.269–0.879	-	2.80	1.68–8.50	-
*L. trifolii*	420	15.05	4	0.005	2.68+0.69	39.52	17.03–67.91	70.0	118.70	68.76–1441	42.4
						Cyromazine					
*L. sativae*	420	13.12	4	0.011	2.33+0.41	12.62	7.07–20.05	-	44.79	26.62–147.89	-
*L. trifolii*	420	4.99	4	0.29	1.77+0.18	71.81	57.25–88.48	5.7	395.14	290.20–606.76	8.8

a SR – Susceptibility ratio  =  LC_i_ (*L. trifolii*)*/*LC_i_ (*L. sativae*).

### Mixed Species Experiments

**Figure 1:**
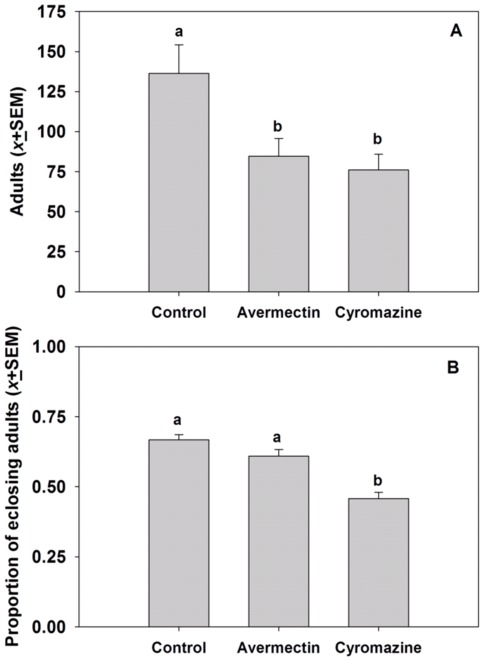
The effects of avermectin (4.5 ppm) and cyromazine (46.8 ppm) on the total number of *Liriomyza* spp. adults (A) and the proportion of pupae that eclosed as adults (B) from cowpea plants infested with both *L. sativae* and *L. trifolii.* Treatments marked with different lower case letters are significantly different.

The results showed that the mean numbers of adults that eclosed from the avermectin and cyromazine treatments were significantly lower than for the control treatment (*F*  = 5.67, df  = 2, 6; *P  = *0.04; Fig. 1a); however, there was no significant difference in the mean number of adults that eclosed from the avermectin and cyromazine treatments (*P*>0.50; Fig. 1a). When considering the rate of adult eclosion in the different treatments, there was a significantly lower eclosion rate from puparia recovered from the cyromazine treatment compared with the avermectin and control treatments (*F*  = 25.13, df  = 2, 6, *P*  = 0.0012; Fig. 1b).

**Figure 2:**
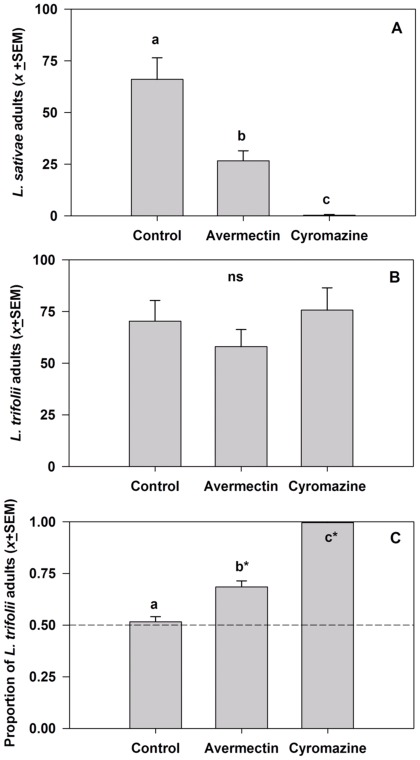
The effects of avermectin (4.5 ppm) and cyromazine (46.8 ppm) on the number of eclosing adults of *Liriomyza sativa* (A) and *L. trifolii* (B), and the proportion of adults that were *L. trifolii* (C). Treatments marked with different lower case letters are significantly different; ns indicates no significant difference among the treatments for that particular variable. For the proportion of adults that were *L. trifolii,* treatments marked with an asterisk (*) indicate the proportion differed significantly from 0.5.

The insecticide treatments had differential effects on the numbers of emerging adults of each species. The numbers of *L. sativae* adults that eclosed differed significantly among the treatments (*F*  = 18.66; df  = 2, 6; *P*  = 0.0027), with significantly more *L .sativae* eclosing in the control treatment than in either the avermectin or cyromazine treatments (Fig. 2a). Almost no *L. sativae* adults eclosed from the cyromazine treatment, whereas an intermediate number eclosed from the avermectins treatment. In contrast, there were no significant differences in the numbers of *L. trifolii* adults from the three different treatments (*F*  = 0.93, df  = 2, 6, P  = 0.45; Fig. 2b). As a result of these interspecific differences in response to the insecticide treatments, there was also a significant treatment effect on the proportions of *L. trifolii* adults among all adults that eclosed (*F*  = 22.01, df  = 2, 6, P  = 0.0017, Fig. 2c). The proportion of *L. trifolii* among the adults that eclosed in the control treatment was 0.52±0.02. This value was not significantly different from a proportion of 0.5 (*t*  = 0.64, P  = 0.55), indicating that in the control treatment the two leafminer species had similar reproductive success. However, *L. trifolii* was the predominant species emerging from plants treated with avermectins, in which over two thirds of the adults were *L. trifolii,* or with cyromazine, in which over 99% of the adults were *L. trifolii* (Fig. 2c).

### Field Plot Experiments

**Figure 3:**
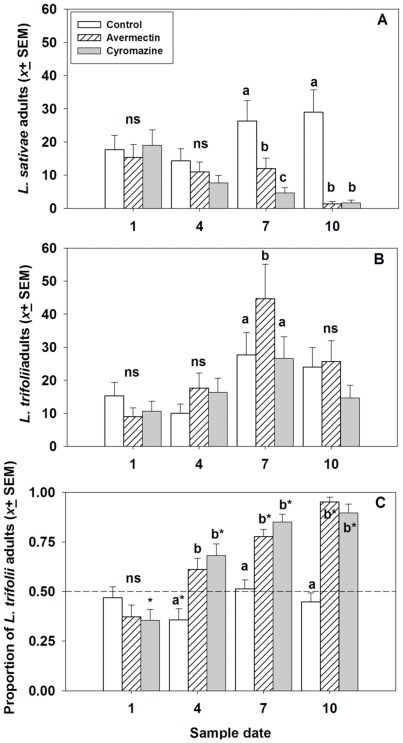
The effects of avermectin (4.5 ppm) and cyromazine (46.8 ppm) applications on the number of eclosing adults of *Liriomyza sativa* (A) and *L. trifolii* (B), and the proportion of adults that were *L. trifolii* (C) from cowpea foliage in field trials conducted in Siling County. Treatments marked with ns are not significantly different; otherwise, treatments marked with different lower case letters are significantly different. For the proportion of adults that were *L. trifolii,* treatments marked with an asterisk (*) indicate the proportion differed significantly from 0.5. Insecticides were applied to plots after collection of leafminer samples on days 1, 4 and 7 of the experiment.

The only *Liriomyza* species recovered in the two field experiments were *L. sativae* and *L. trifolii.* At Siling County, the number of *L. sativae* adults that eclosed differed among the treatments on the last two sample dates (days 7 and 10 of the experiment), which were the sample dates that followed the second and third insecticide applications. Both avermectin and cyromazine treatments resulted in significantly fewer *L. sativae* eclosing compared with the control treatment (Table 2; Fig. 3a). In contrast, the insecticide treatments had no effect on the number of *L trifolii* adults that eclosed (Table 2; Fig. 3b). The percentage of *L. trifolii* adults that eclosed was not different from the percentage of *L. sativae* on the first sample, which was collected before any insecticide applications, but subsequent collections from the avermectin and cyromazine treatments showed there were significantly greater percentages of *L. trifolii* than *L. sativae* adults over the course of the experiment (Table 2; Fig. 3c).

**Table 2:** Analysis of variance (ANOVA) results for insecticide treatment effects on numbers of *L. sativae* and *L. trifolii* adults that eclosed, and the proportion of *L. trifolii* adults from collections made over 10 days of the experiment.

			*L. sativae*		*L. trifolii*		Proportion of*L. trifolii* Adults	
Sample Date	Treatment df	Error df	*F*	*P*	*F*	*P*	*F*	*P*
				Siling County				
1	2	18	0.19	0.83	0.99	0.39	1.27	0.30
4	2	18	1.30	0.30	1.25	0.31	8.00	0.0033
7	2	18	9.18	0.0018	1.47	0.26	15.98	0.0001
10	2	18	23.65	<.0001	1.40	0.27	24.67	<.0001
				Xingtang County				
1	2	18	0.59	0.56	0.09	0.91	0.19	0.83
4	2	18	16.19	<.0001	0.93	0.41	10.87	0.0008
7	2	18	22.53	<.0001	6.38	0.008	49.39	<.0001
10	2	18	38.80	<.0001	1.90	0.18	30.76	<.0001

Insecticide treatments were applied following sample collections on days 1, 4 and 7.

**Figure 4:**
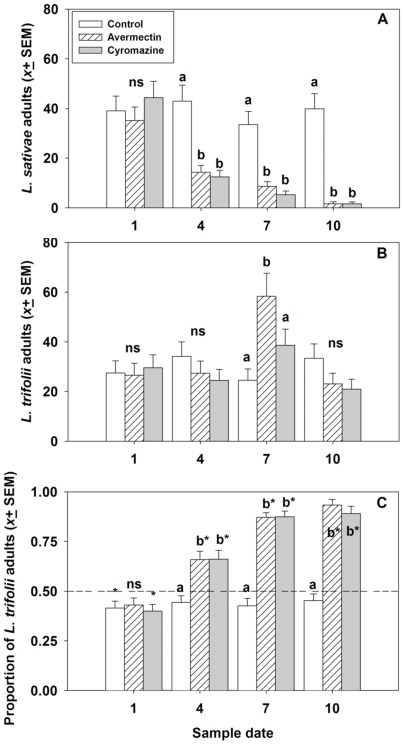
The effects of avermectin (4.5 ppm) and cyromazine (46.8 ppm) applications on the number of eclosing adults of *Liriomyza sativa* (A) and *L. trifolii* (B), and the proportion of adults that were *L. trifolii* (C) from cowpea foliage in field trials conducted in Xingtang County. Treatments marked with ns are not significantly different; otherwise, treatments marked with different lower case letters are significantly different. For the proportion of adults that were *L. trifolii,* treatments marked with an asterisk (*) indicate the proportion differed significantly from 0.5. Insecticides were applied to plots after collection of leafminer samples on days 1, 4 and 7 of the experiment.

Results of the field trial at Xingtang County were similar to those of the field trial at Siling County. The numbers of *L. sativae* adults that eclosed differed among the treatments on all but the first sample date, indicating that avermectin and cyromazine treatments reduced the abundance of *L. sativae* compared with their abundance in control (Table 2; Fig. 4a). In contrast, the insecticide treatments had no effect on the number of *L trifolii* adults that eclosed (Table 2; Fig. 4b). On day 7 of the experiment, there was an unexpectedly high number of *L. trifolii* recovered from the avermectin treatment, which may have been an artifact of a difference in sampling intensity on that day. However, those results did not alter the results for insecticide effects on relative species abundance. The differential response to insecticide treatments led to changes in the composition of the leafminer complex over time. The percentage of individuals of *L. trifolii* (41.5±2.0%) was significantly less than the percentage of *L. sativae* (58.5±2.0%) on the first sample, which was collected before any insecticide applications. However, the three subsequent collections, which followed each of the insecticide applications, showed there was a significantly greater percentages of *L. trifolii* than *L. sativae* adults eclosing from the avermectin and cyromazine treatments (Table 2; Fig. 4c). The percentage of *L. trifolii* adults in the control treatment ranged from 42.6 to 45.3% over the final three sample dates.

## Discussion

Our results demonstrate that *L. trifolii* is significantly more tolerant to avermectin and cyromazine than is *L. sativae,* and these differences could be driving the displacement of *L. sativae* by *L. trifolii.* Although *L. sativae* may have inherent advantages in reproductive success over *L. trifolii* [6], [14], interspecific interactions between these pest species must be viewed within the context of their exposure to insecticides. Avermectin and cyromazine have been used for leafminer management for approximately 30 years. These are the most commonly and widely used insecticides for leafminer management throughout China, and they constitute more than 95% of the insecticide use against leafminers on Hainan Island [10]. Consequently, populations of both species are likely to have experienced extensive, repeated insecticide treatments before and after their introduction to China. The populations used in our lethal concentration and mixed species trials were collected from the same area of Hainan Province and thus the populations would have experienced similar insecticide exposures before the initiation of our experiments. Likewise, the field trials were conducted in areas where *L. trifolii* had only recently invaded and the two species still co-occur, and thus would have similar exposure to insecticides.

Although we did not attempt to assess the resistance status of the test populations, the laboratory leaf dip bioassays showed that the species differed greatly in their susceptibilities, with susceptibility ratios of more than 40× for avermectin and more than 5× for cyromazine (based on the ratio of lethal concentrations of *L. trifolii* relative to *L. sativae*). While the leaf dip bioassays indicated that there was a greater difference between the species in their susceptibilities to avermectin than to cyromazine, the mixed species trials showed that cyromazine had a greater impact than avermectin on *L. sativae* survival. Results of the field trials showed that avermectin and cyromazine were similar in their effects on *L. sativae.*

The results of the field trial show how rapidly the demographics of leafminers can change in response to insecticide applications within a crop. Samples collected before the initial insecticide applications indicate that the two species were coexisting in near equal proportions. However, immediately after insecticide applications began, the proportion of *L. trifolii* in the samples increased. These changes were driven by the susceptibility of *L. sativae* to avermectin and cyromazine, as indicated by the lower abundance of *L. sativae* in those treatments relative to the control, combined with the high tolerance of *L. trifolii* to the rates of insecticides used, as indicated by the lack of difference in *L. trifolii* abundance among the treatments over time.

Comparative toxicity data often have indicated that *L. trifolii* populations are more tolerant to most insecticides than are *L. sativae* populations [2]. Such differences have been implicated in the displacement of *L. sativae* by *L. trifolii* in California [3]. One exception to this general trend has recently been identified in Japan. Tokumaru et al. [15] found that certain populations of *L. sativae* were somewhat more tolerant than an allopatric population of *L. trifolii* to several different classes of insecticides. Although these differences may reflect differences in local insecticide practices rather than inherent species differences, they may contribute to the displacement of *L. trifolii* by *L. sativae* in Japan. If *L. sativae* is not at a disadvantage in terms of insecticide susceptibility, its greater reproductive success may then have allowed it to displace *L. trifolii* [6]. These findings are supported by results of earlier studies in which the predominant leafminer species in untreated lettuce (*Lactuca sativa* L.) in Arizona (USA) was *L. sativae,* but *L. trifolii* was the most abundant species present in commercial fields that received intense insecticide applications [14].

In the present study, had *L. sativae* and *L. trifolii* been similar in their susceptibilities, we would have expected lower abundances of both species in the insecticide treatments relative to the control treatments in the mixed species tests and field trials. However, it was clear that, at the tested rates, *L. sativae* was extremely susceptible, but *L. trifolii* was not affected by either avermectin or cyromazine. These results would be expected based on the leaf dip bioassays because the recommended field rates we used were greater than the LC_90_ values for *L. sativae* but considerably lower than LC_90_ values for *L. trifolii* (Table 1).

Differential susceptibility to insecticides has been linked with changes in the demographics of other pest complexes. For example, the spirea aphid, *Aphis spireacola* Patch has displaced *A. pomi* De Geer as the predominant aphid pest of apple (*Malus domestica* Borkh.) in the eastern and northwestern USA [16]–[18]. Studies conducted in both geographic regions have shown that *A. spireacola* is significantly less susceptible than *A. pomi* to a range of commonly used aphicides. *Aphis pomi* is only able to persist at low levels in the apple system because of differences in the species dispersal behavior [18]. Similarly, the displacement of the B biotype of *Bemisia tabaci* (Gennadius) by the Q biotype in many regions where they have both invaded has been attributed to the greater insecticide resistance of the Q biotype [19], [20]. This difference allows the Q biotype to overcome the competitive advantage that the B biotype has in the absence of insecticide pressures [21].

Given the results of our studies and the reported increasing prevalence of *L. trifolii* throughout China, it is likely that pest management programs for leafminers will need to be adjusted. The results of our study point to the need to accurately identify *Liriomyza* species and to develop species-specific management strategies for these pests. Although both species are capable of causing similar damage to host plants [2], *L. trifolii* is often considered the more significant pest because of the greater difficulty in managing it through insecticide use. Given that we found *L. sativae* to be more sensitive than *L. trifolii* to avemectin and cyromazine, changes in population demographics following insecticide applications would be expected to occur. Continued use of either avermectin or cyromazine at the recommended rates could lead to apparent control failures in the field. Although documented cases of resistance to these insecticides have rarely been reported for *Liriomyza* spp. [22], the potential for resistance exists, especially if increased application rates are used. Development of resistance to either insecticide would be problematic because of the lack of alternative insecticides. To help mitigate problems with either species, IPM programs that emphasize the conservation of parasitoids should be implemented. *Liriomyza* species can be regarded as secondary pests with outbreaks most likely to occur following intensive insecticide use that eliminates the parasitoid complex [23]–[25].

## Materials and Methods

### Ethics Statement

No specific permissions were required for these locations or activities. The location is not privately-owned or protected in any way. The field studies did not involve endangered or protected species.

### Insect Strains

Populations of both *L. sativae* and *L. trifolii* were collected from a cowpea, *Vigna unguiculata* L. Walp., field in Sanya, Hainan Province, in 2007. Both populations were subsequently reared separately on cowpea plants in the absence of insecticides at the Sanya Experiment Station under controlled conditions (26±2°C, 70±10% RH, 14∶10 h light: dark). Before initiation of the experiments in 2011, 25 individuals from each population were identified to species, and the results showed that both populations only contained individuals of one species.

### Larval Bioassays

The bioassay technique used to determine differential susceptibility of *L. sativae* and *L. trifolii* to avermectin and cyromazine was similar to the larval bioassay method described by Ferguson [22]. For each leafminer species, 20 young (10–14-d old) cowpea plants were caged and exposed to several hundred 3–4 d-old flies for an oviposition access period of 2–4 h, which allowed for cohorts of similarly aged larvae to be used in the bioassays. After the oviposition access period, plants were removed and held in a leafminer-free greenhouse for 72 h to allow eggs to hatch and larvae to begin to develop. The numbers of small mines (∼5 mm in length, corresponding to young 2^nd^ instars) present were counted under 10× magnification. Plants were then divided into groups so that there were equal numbers of larvae (n  = 30) to test for each insecticide concentration. The leaves containing 2^nd^ instar leafminers were treated by immersion for 5 s into the appropriate serial dilution of insecticide in distilled water. Control leaves dipped in distilled water only. Seven concentrations of each insecticide were used for each species. After treatment, plants were held in a greenhouse (28°C and ambient light) for 2–3 d to allow larval development to continue. When it became apparent that larvae from the control plants were ready to exit their mines, all leaves were excised and placed on plastic frames in containers (25×25×20 cm) to collect puparia. Puparia were counted and held in glass scintillation vials for adult emergence. Adults were counted upon eclosion.

The data from the insecticide toxicity determinations were subjected to probit analysis using POLO-PC (LeOra Software, Berkeley, CA) after correcting for control mortality with Abbott’s formula [26]. Control mortality was ≤5%. Lethal concentration values (LC) were compared based on their 95% confidence limits, with non-overlapping intervals used to establish significance. The susceptibility ratios for the two species were calculated by dividing the LC estimates of *L. trifolii* by the corresponding LC estimates for *L. sativae.*

### Mixed Species Experiments

The recommended field rates of avermectin (4.5 ppm [A.I.]) and cyromazine (46.8 ppm [A.I.]) were selected for use to explore the effects of differential insecticide susceptibility on species exclusion when both species infest a plant. To obtain plants infested with both leafminer species, 100 male adults and 100 female adults (all 3- to 5-day-old virgins) of each *L. sativae* and *L. trifolii* were added to a cage (200×200×200 cm) containing 20 young cowpea plants (10–14-d-old). Seventy-two h later, plants were sprayed to runoff with one of three insecticide treatments: avermectin (4.5 ppm), cyromazine (46.8 ppm) or a control (distilled water). When it became apparent that larvae in the control plants were ready to exit their mines, all leaves were excised and placed on plastic containers (40×40×20 cm) to collect puparia. Puparia were counted and held in glass scintillation vials for adult emergence. Adults were counted upon eclosion and identified to species. These trials were conducted in a greenhouse at the Sanya Experiment Station. All treatments were replicated three times.

Total numbers of puparia and numbers of adults of each species eclosing in each treatment were compared by employing a generalized linear model with a negative binomial distribution and a log link [27]. The proportion of adults eclosing from puparia and the proportion of *L. trifolii* among the eclosed adults were then compared by employing generalized linear models, incorporating a binomial distribution of the response data and a logit link. Treatment means were separated using the least squares means option [27].

### Field Experiments

Based on recent surveys conducted by Gao et al. [10], *L. trifolii* has become the dominant species and *L. sativae* has become rare in Hainan Province in recent years. Therefore, it was not possible to find a suitable site for field experiments in Hainan in 2011. Consequently, field experiments were conducted at Xingtang and Siling Counties, located in Guangzhou City, Guangdong Province in 2011, where *L. sativae* and *L. trifolii* are known to still co-occur (Gao et al., unpublished data). Cowpea was planted in both counties on 10 March 2011 at sites where *L. trifolii* and *L. sativae* were observed occurring simultaneously. Both field experiments used a randomized complete block design with three replications of each of three treatments. The three treatments were avermectin applied at 4.5 ppm [A.I.], cyromazine applied at 46.8 ppm [A.I], and distilled water. The hand-pump type sprayer used to apply treatments was calibrated to deliver 1300 L/ha at 20–30 kPa, with 90 µm openings in the nozzles. A 3 m space was set up between plots to minimize insecticide contamination among treatment plots. Each plot was approximately 0.004 ha and seeded at a rate expected to produce 1500 plants per plot, as is common in this farming system. Cowpea was maintained using the standard agronomic practices of the local areas, and no other insecticides were used throughout the season.

Sampling for leafminers and insecticide applications began at the end of April, approximately 7 wk after sowing. Sampling for leafminers was conducted on days 1, 4, 7 and 10 of the experiments. Insecticides were applied after leafminer samples were collected on day 1, 4 and 7 of the experiment. Sampling and insecticide applications began at the end of April. Five sites were randomly chosen within each plot on each sampling date, with a total of 50–200 leaves containing late-instar larvae collected per plot per date. Leaves were excised and placed on plastic containers (40×40×20 cm) to collect puparia. Puparia were held in glass scintillation vials for adult emergence. Adults were counted and identified to species.

For these field experiments, numbers of adults of each species eclosing in each treatment were compared by employing a generalized linear model with a negative binomial distribution and a log link [27]. The proportion of *L. trifolii* among the eclosed adults were then compared by employing a generalized linear model, incorporating a binomial distribution of the response data and a logit link. Because of the repeated nature of the data, analyses were conducted as repeated measures ANOVAs. Because of significant date by treatment interactions, simple effects for the insecticide treatments on each sample date were tested by slicing treatment effects by sample date [27]. Treatment means were separated using the least squares means option [27].
